# Tracheoinnominate Artery Fistula

**DOI:** 10.21980/J8K05R

**Published:** 2021-07-15

**Authors:** Emily M Tarver, Anna A Lerant, Jeffrey D Orledge, Benjamin P Stevens, Gina D Jefferson

**Affiliations:** * University of Mississippi Medical Center, Department of Emergency Medicine, Jackson, MS; ^ University of Mississippi Medical Center, Department of Anesthesiology, Jackson, MS; ‡ University of Mississippi Medical Center, Department of Otolaryngology, Jackson, MS

## Abstract

**Audience:**

This simulation provides training for emergency medicine residents in the stepwise management of a patient who presents with bleeding from a tracheoinnominate artery fistula. Additional learners who might benefit from this simulation are otolaryngology and general surgery residents as well as critical care fellows.

**Introduction:**

Hemorrhage from a tracheoinnominate artery fistula (TIAF) is a rare but life-threatening complication in a patient with a recent tracheostomy. This complication occurs in 0.7% of tracheostomy patients with a mortality of 50–70%.^1^ Seventy-five percent of patients with a TIAF will present within the first three weeks of surgery and 50% of patients will present with a sentinel bleed that briefly resolves.^1^ Key elements of a history and exam that should raise a provider’s concern for this diagnosis include a recent tracheostomy (within the last 4 weeks), a percutaneous tracheostomy, prior radiation, chronic steroid use, a neck or chest deformity or a sentinel bleed.^2^ Survival from a TIAF hinges upon emergent, operative repair by an otolaryngologist and cardiothoracic surgeon. Cuff hyperinflation and the Utley Maneuver are critical bedside interventions to temporize this massive bleed and stabilize the patient for definitive, operative repair.

**Educational Objectives:**

By the end of this simulation, learners will be able to: 1) perform a focused history and physical exam on any patient who presents with bleeding from the tracheostomy site, 2) describe the differential diagnosis of bleeding from a tracheostomy site, including a TIAF, 3) demonstrate the stepwise management of bleeding from a suspected TIAF, including cuff hyperinflation and the Utley Maneuver, 4) verify that definitive airway control via endotracheal intubation is only feasible in the tracheostomy patient when it is clear, upon history and exam, that the patient can be intubated from above, 5) demonstrate additional critical actions in the management of a patient with a TIAF, including early consultation with otolaryngology and cardiothoracic surgery as well as emergent blood transfusion and activation of a massive transfusion protocol.

**Educational Methods:**

This case was written with a modified, low-fidelity manikin, traditionally used for training in nasogastric tube placement and tracheostomy care. We modified this manikin to simulate a hemorrhage from the tracheostomy site.^3^ The patient in our case had a history of laryngeal cancer, and thus we occluded his larynx for this simulation. As a result of this obstruction, he was unable to be intubated from above. We provided confederates, a bedside nurse and family member, to assist the learners throughout the case. We also utilized a simulation technician to operate dynamic vital signs on a simulated cardiac monitor. It would be technically challenging to adapt this case to a high-fidelity simulator due to potential for damage of the internal electrical elements by the large amount of artificial blood from the tracheostomy tube. However, a mechanical pump provided a useful means of active bleeding in this low-fidelity manikin.

**Research Methods:**

We provided a pre- and post-simulation questionnaire for the 33 emergency medicine residents who participated in this simulation. There were 11 residents from each of the PGY-1, PGY-2 and PGY-3 year-groups. Thirty-two residents (97%) completed the pre-survey and 33 residents (100%) completed the post-survey. For our questions, we used a 5-point Likert Scale to assess a resident’s knowledge of the learning objectives within this simulation.

**Results:**

Responses from our pre- and post- survey indicated a significant improvement in knowledge about a tracheoinnominate artery fistula as well as the general management of tracheostomy complications in the emergency department.

**Discussion:**

This simulation is a useful educational tool for instructing emergency medicine residents on optimal management of tracheostomy emergencies such as a TIAF. The interprofessional teaching by an emergency medicine attending and mid-level (PGY-3) otolaryngology resident allowed for a richer and more detailed discussion during the debriefing. Throughout the case, the emergency medicine attending played the role of a bedside nurse and offered supportive, clinical cues when bleeding recurred. The otolaryngology resident played the role of a family member and offered helpful cues during the history and exam portion of the case. Following the case, both content experts provided useful clinical insight during the debriefing. If staffing availability permits, it might be advantageous to use additional simulation-trained personnel to play the roles of the nurse and family member, thus allowing the emergency medicine attending and otolaryngology content experts to simply view the case from the control room and perform the debriefing.

**Topics:**

Tracheostomy, surgical airway, tracheoinnominate artery fistula, bleeding from tracheostomy site, complications with tracheostomies, hemorrhagic shock.

## USER GUIDE

[Table t1-jetem-6-3-s62]

**Table t1-jetem-6-3-s62:** 

**List of Resources:**	
Abstract	62
User Guide	64
Instructor Materials	67
Operator Materials	73
Debriefing and Evaluation Pearls	77
Simulation Assessment	81

**Learner Audience:**

Interns, Junior Residents, Senior Residents, Critical Care Fellows

**Time Required for Implementation:**

Instructor Preparation: 1 hour

Time for case: 15–20 minutes

Time for debriefing: 30–40 minutes

**Recommended Number of Learners per Instructor:**

6:2

**Topics:**

Tracheostomy, surgical airway, tracheoinnominate artery fistula, bleeding from tracheostomy site, complications with tracheostomies, hemorrhagic shock.

**Objectives:**

By the end of this simulation session, learners will be able to:

1. Perform a focused history and physical exam on any patient who presents with bleeding from the tracheostomy site.
2. Describe the differential diagnosis of bleeding from a tracheostomy site, including a TIAF.
3. Demonstrate the stepwise management of bleeding from a suspected TIAF, including cuff hyperinflation and the Utley Maneuver.
4. Verify that definitive airway control via endotracheal intubation is only feasible in the tracheostomy patient when it is clear, upon history and exam, that the patient can be intubated from above.
5. Demonstrate additional critical actions in the management of a patient with a TIAF, including early consultation with otolaryngology and cardiothoracic surgery as well as emergent blood transfusion and activation of a massive transfusion protocol.

### Linked objectives and methods

Emergency medicine providers commonly evaluate patients with complications from their tracheostomy. Most of these patients remain stable and are ultimately discharged from the emergency department. A tracheoinnominate artery fistula (TIAF) represents a rare but life-threatening complication from a tracheostomy procedure, usually within the first month. Other emergent complications from a tracheostomy are an acute obstruction, site infection or displacement of the tube. For this reason, the provider must rapidly perform a focused history and exam on any patient who presents with bleeding or other complications from a tracheostomy site (objective 1). When the patient in this simulation reports “½ cup” of bleeding from the tracheostomy tube, the evaluating providers needs to maintain an accurate differential diagnosis of bleeding from a tracheostomy site (objective 2). They should be particularly concerned about a TIAF, given that the tracheostomy is only 2 weeks old. After completion of a thorough history and physical exam, the simulation operator produces a series of coughs through the overhead speaker. This will cue the nurse at the bedside to discreetly activate the arterial pump, and the patient will begin to hemorrhage from the tracheostomy. At this point in the case, the providers must rapidly act to stop the bleed (objective 3). They must replace the uncuffed tracheostomy tube with a cuffed tracheostomy or endotracheal tube and hyperinflate the cuff. Bleeding temporarily stops but when it restarts, they must slowly pull back the tube and exert anterior pressure on the trachea. Again, bleeding will temporarily stop but when it restarts, the provider must demonstrate the Utley Maneuver. To perform this maneuver, the provider must deflate the cuff, place a gloved finger in the stoma and apply direct pressure between the bleeding vessel and posterior superior sternal wall. If providers are unable to fit their finger in the stoma with the cuffed tube, they may replace the cuffed tube with a smaller, uncuffed tube and reattempt the Utley Maneuver. The provider must demonstrate knowledge of each of these steps if bleeding recurs prior to surgical repair. Recurrent bleeding from the tracheostomy in this case portends a potentially failed airway, and the provider must only consider intubation from above if a thorough history and exam has deemed this a feasible endeavor (objective 4). In the case of this patient, his laryngeal cancer history made him an unsafe candidate for a traditional intubation attempt. This is a critical piece of any history for a patient who presents with potential airway compromise at the tracheostomy site. Rapid and early mobilization of otolaryngology, cardiothoracic surgery and the operating room team is imperative in this case. It is reasonable for the provider to consult with a specialist for any amount of bleeding greater than 10mL from the tracheostomy tube.^4^ Due to repeated hemorrhage, despite multiple bedside maneuvers, transfusion of emergent-release blood and activation of a massive transfusion protocol are additional, life-saving maneuvers as this patient is transferred to the operating room (objective 5). Inattention to any of these objectives might be catastrophic for an actual patient who presents with a TIAF and are thus detailed in this simulation.

### Recommended pre-reading for instructor

- Bontempo LJ, Manning SL. Tracheostomy Emergencies. *Emerg Med Clin North Am*. 2019;37(1):109–119. doi:10.1016/j.emc.2018.09.010
- McGrath BA, Bates L, Atkinson D, Moore JA. Multidisciplinary guidelines for the management of tracheostomy and laryngectomy airway emergencies. *Anaesthesia*. 2012;67:1025–41. doi:10.1111/j.1365-2044.2012.07217.x.
- Weingart S. Podcast 195 – Management of Tracheostomy (Trach) and Laryngectomy Emergencies. EMCrit. March 20, 2017. Accessed August 15, 2021. At: https://emcrit.org/emcrit/tracheostomy-emergencies/
- Gupta V, Swaminathan A. CORE EM: Common Tracheostomy Issues. emDOCs.net. June 28, 2019. Accessed September 10, 2020. http://www.emdocs.net/core-em-commontracheostomy-issues/
- Bryant CD. Complications of Airway Devices. Meckler GD, Stapczynski J, Cline DM, et al, eds. *Tintinalli’s Emergency Medicine: A Comprehensive Study Guide*. 9^th^ ed. McGraw-Hill Medical. Accessed March 22, 2021. At: https://accessemergencymedicine.mhmedical.com/content.aspx?bookId=2353&sectionId=221180267

### Learner responsible content

- Bontempo LJ, Manning SL. Tracheostomy Emergencies. *Emerg Med Clin North Am*. 2019;37(1):109–119. doi:10.1016/j.emc.2018.09.010
- McGrath BA, Bates L, Atkinson D, Moore JA. Multidisciplinary guidelines for the management of tracheostomy and laryngectomy airway emergencies. *Anaesthesia*. 2012;67:1025–41. doi:10.1111/j.1365-2044.2012.07217.x
- Weingart S. Podcast 195 – Management of Tracheostomy (Trach) and Laryngectomy Emergencies. EMCrit. March 20, 2017. Accessed August 15, 2021. At: https://emcrit.org/emcrit/tracheostomy-emergencies/
- Gupta V, Swaminathan A. CORE EM: Common Tracheostomy Issues. emDOCs.net. June 28, 2019. Accessed September 10, 2020. At: http://www.emdocs.net/core-em-commontracheostomy-issues/
- Bryant CD. Complications of Airway Devices. In: Tintinalli JE, Ma O, Yealy DM, et al, eds. *Tintinalli’s Emergency Medicine: A Comprehensive Study Guide*, 9^th^ ed. McGraw-Hill Medical. Accessed March 22, 2021. At: https://accessemergencymedicine.mhmedical.com/content.aspx?bookId=2353&sectionId=221180267

### Results and tips for successful implementation

We offered this simulation to emergency medicine residents in small groups with 5 to 6 learners per group. Members of each group went through the entire case together. A more ideal number of participants might be 1 or 2 residents per group, to offer each participant a more hands-on experience. We had a simulated patient monitor, and a simulation operator made dynamic changes to the monitor during the case. We had an emergency medicine (EM) attending who played the role of the emergency department nurse and a mid-level, otolaryngology (ENT) resident who played the role of the patient’s family member during the case. This latter role is very helpful to boost the fidelity of the case because patients with tracheostomy tubes often have limited or no phonation and an additional historian is commonly needed. The ED nurse gave the learners a brief triage history upon starting the case, and then the learners assumed full management of the patient.

The EM attending and ENT resident performed a bedside debriefing after the case. One benefit of a bedside debriefing was the hands-on access to the manikin and supplies to directly review any critical steps that were missed during the simulation. We allowed 40 minutes per group for the entire simulation but would allot an entire hour for this exercise in the future. This case raised many thoughtful questions regarding management of a tracheoinnominate artery fistula and general tracheostomy care in the ED; ample time should be allowed for this discussion during the debrief. Review of tracheostomy equipment such as cuffed and uncuffed devices, their endotracheal tube counterparts, basic tracheal suctioning, and best ways to both oxygenate and ventilate a tracheostomy patient are better reviewed on the manikin during a bedside debriefing. We provided a 3D anatomic model of the brachiocephalic trunk, in scaled proximity to cardiac and pulmonary structures, to reinforce the pathophysiology of a TIAF. We also highly recommend having both an emergency medicine and otolaryngology content expert as co-facilitators for this case. As an interdisciplinary offering, both perspectives are extremely helpful during the debriefing. To avoid the possibility of revealing the case, we did not provide any prereading to learners before this case. In retrospect, this would have greatly reinforced the learning during and after this case. Along similar lines, a post-simulation reading list would be a helpful resource for additional review.

Our pre- and post-survey results revealed that the emergency medicine residents who completed this simulation significantly improved their knowledge in the management of this rare but life-threatening condition. When asked about their familiarity with the Utley Maneuver, 28% of residents (9/32) responded “*agree*” or “*strongly agree*” before the simulation, and 91% (30/33) of residents responded “*agree*” or “*strongly agree*” after the simulation. When asked about their knowledge of additional methods to curb bleeding from a tracheostomy site, 25% of residents (8/32) responded “*agree*” or “*strongly agre*e” before the simulation, while 94% of residents (31/33) responded “*agree*” or “*strongly agree*” after the simulation. When asked about baseline knowledge of a differential diagnosis of bleeding from a tracheostomy site, 22% of residents (7/32) responded “*agree*” or “*strongly agree*” before the simulation, and 94% of residents (31/33) responded “*agree*” or “*strongly agree*” after the simulation. Responses from our pre- and post-survey indicated a significant improvement in knowledge about tracheoinnominate artery fistula as well as general management of tracheostomy complications in the emergency department.

## Figures and Tables

**Table t2-jetem-6-3-s62:** 

**Vitals:**	Heart Rate (HR) 75	Blood Pressure (BP) 135/70	Respiratory Rate (RR) 14
	Temperature (T) 37.0°C	Oxygen Saturation (O2Sat) 96% on room air	

**Table t3-jetem-6-3-s62:** SIMULATION EVENTS TABLE:

Minute (state)	Participant action/trigger	Patient status (simulator response) & operator prompts	Monitor display (vital signs)
0:00 (Baseline)	Attach patient to cardiac monitor and pulse oximetry. Attach an automated blood pressure cuff. Perform a complete history and physical exam Obtain IV access and order lab work: complete blood count (CBC), basic metabolic panel (BMP) coagulation studies (PT/PTT) and type and screen	Patient is stable and comfortable appearing on initial assessment. Nurse may ask if it is ok to put him back in the waiting room until a bed becomes available. Due to concern for a sentinel bleed from a TIAF this would be unsafe, and the provider should immediately try and get him into an exam room.	T 37.0°C HR 75 BP 135/70 RR 14 O2 96%
5:00	Nurse at bedside to discreetly turn on the pump to activate bleeding from the tracheostomy site when the simulation operator sounds a loud cough from the patient.	Bleed will not stop with any attempted suctioning. Participants should replace the uncuffed tracheostomy tube with a cuffed tracheostomy tube of similar size and hyperinflate. A bougie or other exchange catheter is optional for use. If a hyperinflated, cuffed tube is not placed, bleeding will continue. Learner should voice concern for a TIAF and consult emergently with ENT, CT, Surgery and the OR Team for definitive repair. If learner does not consult early with specialties, the family member states that his otolaryngologist was very concerned and asked to be called immediately upon arrival to the ED, prompting a consultation for next steps.	**Hyperinflated, Cuffed tube:** HR 95 BP 150/80 RR 20 O2 92% on Room Air **No cuffed tube:** HR 110 BP 110/65 RR 24 02 88% on Room Air
8:00	Bleeding briefly stops with placement of a hyperinflated, cuffed tube. Several minutes later, the bleeding restarts.	Patient appears more anxious. Nurse informs the provider that the OR team is on the way. Provider must slowly withdraw the tube while exerting pressure against the anterior trachea. This maneuver will stop the bleeding again. If this maneuver is not performed, then bleeding continues. The ENT consultant may call for a status update and instructs learner on placement of a hyperinflated, cuffed tube, anterior tracheal pressure and the Utley Maneuver, if refractory bleeding, to stabilize before the OR.	**Anterior Pressure:** HR 120 BP 90/60 RR 22 O2 92% **No cuffed tube and/or anterior pressure:** HR 140 BP 80/50 RR 28 02 88% on room air
		Ask bedside nurse to assist with gentle suctioning of blood in tubing as well as supplemental oxygen either by non-rebreather directly over the tracheostomy or use of ambu bag to provide positive pressure ventilations through the tracheostomy. Consider small dose of an anxiolytic such as versed if using positive pressure ventilation. Nurse may suggest the suctioning and supplemental oxygen if not requested.	**Use of suctioning and supplemental 02:** HR: 120 BP:90/60 RR:20 02 Sat: 96% on supplemental 02 **If no suctioning and/or supplemental 02:** HR 118 BP 90/60 RR 24 O2 84% on room air
		The provider should order 2 units of emergent release, packed red blood cells (PRBC) and activate a massive transfusion protocol.	**PRBC:** HR: 100 BP:100/65 RR:18 02 Sat: 96% on supplemental 02 **No PRBC:** HR: 130 BP:80/55 RR:22 02 Sat: 96% on supplemental 02
10:00	Bleeding briefly stops by withdrawing the tube slightly. It then restarts several minutes later.	Patient still appears anxious but remains alert. Provider performs the Utley Maneuver by placing one gloved finger inside the tracheostomy site, attempting to compress the bleeding vessel between the finger and posterior, superior sternum. The provider must maintain this position until definitive repair in the OR. If the provider does not perform the Utley Maneuver and maintain this intervention until definitive, operative repair, then the patient will continue to hemorrhage from the tracheostomy site. ENT consultant may arrive and perform this maneuver if learner does not.	**Utley:** HR 118 BP 95/64 RR 22 O2 92% on supplemental 02 **No Utley** HR 140 BP 80/50 RR 28 02 88% on supplemental 02
(Case Completion)	Patient transport to the OR with provider holding direct pressure via Utley Maneuver	Ongoing transfusion of packed red blood cells (PRBC) with massive transfusion protocol (MTP) in place and additional blood products on standby in the OR.	**PRBC/MTP:** HR 110 BP 100/70 RR 20 O2 94% (non-rebreather mask or positive pressure ventilation with ambu bag) **If NO blood transfusion:** HR 140 BP 80/50 RR 28 02 92% on supplemental 02

**Table t4-jetem-6-3-s62:** 

**Critical Actions:** Perform a detailed history and exam of a patient who presents with bleeding from the tracheostomy site. This should include clarification of whether the patient can be intubated from above. Order one or two, large-bore peripheral IVs and bloodwork, including type and screen, complete blood count (CBC), basic metabolic panel (BMP) and coagulation panel. When the rebleed occurs, apply gentle suction and replace the uncuffed tracheostomy tube with a cuffed tracheostomy tube (or cuffed endotracheal tube) of similar size and hyperinflate the cuff. Recognize this is likely a tracheoinnominate artery fistula and emergently consult with otolaryngology, cardiothoracic surgery and the operating room team. If rebleed occurs, gently pull back the tube and exert anterior pressure on the trachea. If rebleed occurs, deflate the cuff and perform the Utley Maneuver. The providers who perform this maneuver must not move their finger until the patient is in the operating room and under the care of the surgical team. If the providers are unable to fit their finger in the stoma, they may reattempt after replacement of the cuffed with an uncuffed tube that is one size smaller. When the patient becomes hemodynamically unstable from recurrent bleeding, the provider must order two units of emergent-release blood and activate a massive transfusion protocol.	0:00

**Table t5-jetem-6-3-s62:** 

	Milestone	Did not achieve level 1	Level 1	Level 2	Level 3
1	**Emergency Stabilization (PC1)**	□ Did not achieve Level 1	□ Recognizes abnormal vital signs	□ Recognizes an unstable patient, requiring intervention Performs primary assessment Discerns data to formulate a diagnostic impression/plan	□ Manages and prioritizes critical actions in a critically ill patient Reassesses after implementing a stabilizing intervention
2	**Performance of focused history and physical (PC2)**	□ Did not achieve Level 1	□ Performs a reliable, comprehensive history and physical exam	□ Performs and communicates a focused history and physical exam based on chief complaint and urgent issues	□ Prioritizes essential components of history and physical exam given dynamic circumstances
3	**Diagnostic studies (PC3)**	□ Did not achieve Level 1	□ Determines the necessity of diagnostic studies	□ Orders appropriate diagnostic studies. Performs appropriate bedside diagnostic studies/procedures	□ Prioritizes essential testing Interprets results of diagnostic studies Reviews risks, benefits, contraindications, and alternatives to a diagnostic study or procedure
4	**Diagnosis (PC4)**	□ Did not achieve Level 1	□ Considers a list of potential diagnoses	□ Considers an appropriate list of potential diagnosis May or may not make correct diagnosis	□ Makes the appropriate diagnosis Considers other potential diagnoses, avoiding premature closure
5	**Pharmacotherapy (PC5)**	□ Did not achieve Level 1	□ Asks patient for drug allergies	□ Selects an medication for therapeutic intervention, consider potential adverse effects	□ Selects the most appropriate medication and understands mechanism of action, effect, and potential side effects Considers and recognizes drug-drug interactions
6	**Observation and reassessment (PC6)**	□ Did not achieve Level 1	□ Reevaluates patient at least one time during case	□ Reevaluates patient after most therapeutic interventions	□ Consistently evaluates the effectiveness of therapies at appropriate intervals
7	**Disposition (PC7)**	□ Did not achieve Level 1	□ Appropriately selects whether to admit or discharge the patient	□ Appropriately selects whether to admit or discharge Involves the expertise of some of the appropriate specialists	□ Educates the patient appropriately about their disposition Assigns patient to an appropriate level of care (ICU/Tele/Floor) Involves expertise of all appropriate specialists
9	**General Approach to Procedures (PC9)**	□ Did not achieve Level 1	□ Identifies pertinent anatomy and physiology for a procedure Uses appropriate Universal Precautions	□ Obtains informed consent Knows indications, contraindications, anatomic landmarks, equipment, anesthetic and procedural technique, and potential complications for common ED procedures	□ Determines a back-up strategy if initial attempts are unsuccessful Correctly interprets results of diagnostic procedure
20	**Professional Values (PROF1)**	□ Did not achieve Level 1	□ Demonstrates caring, honest behavior	□ Exhibits compassion, respect, sensitivity and responsiveness	□ Develops alternative care plans when patients’ personal beliefs and decisions preclude standard care
22	**Patient centered communication (ICS1)**	□ Did not achieve level 1	□ Establishes rapport and demonstrates empathy to patient (and family) Listens effectively	□ Elicits patient’s reason for seeking health care	□ Manages patient expectations in a manner that minimizes potential for stress, conflict, and misunderstanding. Effectively communicates with vulnerable populations, (at risk patients and families)
23	**Team management (ICS2)**	□ Did not achieve level 1	□ Recognizes other members of the patient care team during case (nurse, techs)	□ Communicates pertinent information to other healthcare colleagues	□ Communicates a clear, succinct, and appropriate handoff with specialists and other colleagues Communicates effectively with ancillary staff

## INSTRUCTOR MATERIALS

**Case Title:** Tracheoinnominate Artery Fistula

**Case Description & Diagnosis (short synopsis):** A patient with a recent tracheostomy presents to the emergency department (ED) with a sentinel bleed from his tracheostomy tube. The bleeding has initially stopped but the patient subsequently has a massive hemorrhage from the tracheostomy tube after a brief coughing spell. The provider must recognize a tracheoinnominate artery fistula (TIAF) as the likely cause of this hemorrhage and perform stepwise maneuvers to stop the bleed. In addition to hemorrhage control, the provider must consult emergently with otolaryngology, cardiothoracic surgery, and the operative team.

**Equipment or Props Needed:**

- Personal Protective Equipment (gown, gloves, eye, and face protection)
- Low-fidelity nasogastric/tracheostomy task trainer, modified with arterial line pump and tubing: The arterial line pump sits on a mayo stand beside the patient and nurse; the pump and blood reservoir are discreetly covered with a sheet. The arterial line tubing connects to the patient underneath the gown.^3^
- Simulated patient monitor with dynamic vital signs (heart rate, respiratory rate, blood pressure, pulse oximetry)
- Bougie or another airway exchange catheter
- Bedside suction canister with tubing and tracheal suction catheter
- 10 cc syringe for inflation/deflation of cuff
- Crash cart with defibrillator
- Airway cart (including size 5 and 6 cuffed tracheostomy tubes, 4-0 and 5-0 cuffed and uncuffed endotracheal tubes, ambu bag, nonrebreather)
- 2 units of artificial blood and container with additional blood products for massive transfusion protocol

**Confederates needed:**

- Assistant to play nurse at bedside
- Assistant to play family member who provides additional history during the case (this role could be played as voice-over by the simulation operator)
- Simulation Operator to provide “coughing” sounds of patient when bleeding recurs as well as operation of dynamic vital signs on the simulated monitor

**Stimulus Inventory:**

None (nurse may state that x-ray is “pending” as patient is stabilized for the operating room)

**Background and brief information:** The patient presents to a large, academic emergency department with his family member. He reports a moderate amount of bleeding from his tracheostomy tube that he describes as approximately ½ cup in volume. His vitals are stable and bleeding has resolved but there is a visible stain of blood on his shirt from the recent bleed.

**Initial presentation:** The patient initially presents with stable vital signs and history of a recent bleed from his tracheostomy site that has since resolved. A family member is present at the bedside to provide the history.

**How the scene unfolds:** The patient has a history of recurrent laryngeal cancer and a remote history of chemotherapy and radiation treatments. He received a percutaneous, awake tracheostomy 2 weeks ago by an otolaryngologist at the presenting hospital. This was done after he was found to have recurrence of the laryngeal cancer with worsening upper airway obstruction. The patient’s family member is present at the bedside to provide this critical history to the provider. Upon further questioning, the family member denies that the patient has experienced any recent illness or complications until the bleeding episode this morning. They report approximately “½ cup” of blood from the tracheostomy tube that has resolved. He does not take aspirin or other blood thinners. He denies any recent fever or other illness. He denies any difficulty breathing and appears quite comfortable and alert on initial assessment. If asked, the family member adds that the patient’s cancer was blocking his upper airway and he had the tracheostomy performed to breathe better (this cues the learner that the patient is unlikely able to be intubated from above). If the learner consults with the medical record or calls the patient’s otolaryngologist, they will discover the same information.

**Critical actions:**

1. Perform a detailed history and exam of a patient who presents with bleeding from the tracheostomy site. This should include clarification of whether the patient can be intubated from above.
2. Order one or two, large-bore peripheral IVs and bloodwork, including type and screen, complete blood count (CBC), basic metabolic panel (BMP) and coagulation panel.
3. When the rebleed occurs, apply gentle suction and replace the uncuffed tracheostomy tube with a cuffed tracheostomy tube (or cuffed endotracheal tube) of similar size and hyperinflate the cuff.
4. Recognize this is likely a tracheoinnominate artery fistula and emergently consult with otolaryngology, cardiothoracic surgery and the operating room team.
5. If rebleed occurs, gently pull back the tube and exert anterior pressure on the trachea.
6. If rebleed occurs, deflate the cuff and perform the Utley Maneuver. The providers who perform this maneuver must not move their finger until the patient is in the operating room and under the care of the surgical team. If the providers are unable to fit their finger in the stoma, they may reattempt after replacement of the cuffed with an uncuffed tube that is one size smaller.
7. When the patient becomes hemodynamically unstable from recurrent bleeding, the provider must order two units of emergent-release blood and activate a massive transfusion protocol.

**Case Title:** Tracheoinnominate Artery Fistula

**Chief Complaint:** Recent bleed from tracheostomy tube[Table t2-jetem-6-3-s62]

**Primary Survey:**

- **Airway:** Patent, size 6, uncuffed tracheostomy tube, well placed, no active bleeding.
- **Breathing:** Breath sounds clear bilaterally and no accessory muscle use
- **Circulation:** 2+ carotid and femoral pulses, normal heart rate and blood pressure

**History:**

- **History of present illness:** Patient reports ½ cup of bright red blood from his tracheostomy site approximately 30 minutes prior to arrival. He reports no recent illness and feels completely back to baseline with bleeding resolved upon presentation in triage. He had an awake, percutaneous tracheostomy placed at this hospital by his otolaryngologist 2 weeks ago. This was done due to recurrence of his laryngeal cancer with tumor burden in his airway. He does not take blood thinners and reports no complications since this procedure was done.
- **Past medical history:** Recurrent Laryngeal Cancer, Hypertension
- **Past surgical history:** Percutaneous Tracheostomy placement 2 weeks ago at this hospital
- **Medications:** Hydrochlorothiazide
- **Allergies:** No Known Drug Allergies
- **Social history:** Former smoker, quit 10 years ago; denies alcohol or illicit drugs
- **Family history:** None significant

**Secondary Survey/Physical Examination:**

- **General appearance:** Thin-appearing male, alert, no acute distress
- **HEENT:**
- ○ **Head:** normocephalic, atraumatic
- ○ **Eyes:** pupils 4mm equal bilaterally, no discharge
- ○ **Ears:** within normal limits
- ○ **Nose:** within normal limits
- ○ **Throat:** within normal limits
- **Neck:** Size 6, uncuffed tracheostomy tube in mid-anterior neck; no bleeding, swelling or redness
- **Heart:** regular rate, regular rhythm, no murmur
- **Lungs:** normal respiratory rate, clear breath sounds bilaterally
- **Abdominal/GI:** soft, non-tender, non-distended
- **Genitourinary:** within normal limits
- **Rectal:** within normal limits
- **Extremities:** no lower extremity edema, no calf tenderness
- **Back:** within normal limits
- **Neuro:** alert, unable to speak due to destruction of larynx from prior cancer but able to follow all commands with normal motor and sensory findings
- **Skin:** no rash; no abnormal findings around the tracheostomy site
- **Lymph:** bilateral cervical adenopathy
- **Psych:** within normal limits

**Results:**

No lab or imaging results are available as this case unfolds rapidly after presentation from triage and patient is emergently transferred to the operating room while these studies are “pending.”

## OPERATOR MATERIALS

[Table t3-jetem-6-3-s62]

**Diagnosis:**

Tracheoinnominate Artery Fistula

**Disposition:**

Emergent Transport to the OR

## DEBRIEFING AND EVALUATION PEARLS

### Tracheoinnominate Artery Fistula

**Pearls:**

1. This patient appears stable with a recent, sentinel bleed from his tracheostomy site. The learner needs to appreciate that his recent tracheostomy placement makes him higher risk for a tracheoinnominate artery fistula (TIAF). Risk for this complication is highest within the initial four weeks of this procedure.
2. Rule-out a possible bleeding diathesis during a history of the medications. Emergent reversal agents should be considered if the patient is on coumadin or other blood thinners.
3. Early placement of patient, while stable, on a cardiac monitor with pulse oximetry. Early placement of 2 large-bore peripheral IVs in anticipation of potential for deterioration.
4. The learner should attempt early to determine, via ENT consult, review of the operative notes and/or patient history, if the patient can be intubated from above. The learner must also closely monitor the patient’s hemodynamic status due to the potential for serious rebleed upon presentation with a sentinel bleed from a TIAF.
5. Upon consultation with an otolaryngologist and/or cardiothoracic surgeon, in the absence of a rebleed, the provider might consider CT Angiography for more detailed assessment of a possible TIAF. This should only be considered in a stable patient without active bleeding.
6. The learner demonstrates a stepwise approach to hemorrhage from the tracheostomy tube, including placement of a cuffed tube with hyperinflation of the cuff. For continued bleeding, this is followed by a slow withdrawal of the hyper-inflated, cuffed tube, with anterior pressure on the trachea. If bleeding persists, perform the Utley Maneuver (place a gloved finger in the tracheostomy site and exert pressure between the bleeding vessel and the posterior surface of the sternum). If the providers are unable to fit their finger in the ostomy, they may need to replace the cuffed tube with a smaller, uncuffed tube and reattempt. The provider must maintain the Utley Maneuver until definitive, operative repair by ENT and CT Surgery.

**Other debriefing points:**

We implemented several techniques within our case to prevent this patient from dying from hemorrhagic shock. The nurse and family member were able to provide helpful cues if the learner was struggling to control the airway or bleeding. For example, if the learner attempted to intubate the patient from above, the family member might state that he was told this would never be possible because of the size of the cancer in the airway. The nurse might offer suggestions such as replacement of an uncuffed tube with a cuffed tube if the learner is struggling to stop the bleeding. The nurse may even suggest a blood transfusion and supplemental oxygen if the providers are not performing this. The ENT consultant, via phone, can also coach the provider on stepwise maneuvers to temporize bleeding. Prior to the simulation, the facilitators must decide if the confederates will aid the learner in ways that will prevent the patient from ongoing hemorrhage and/or death or whether the learner will act with complete autonomy, at the risk of a potentially fatal outcome. As a group, we decided to offer cues from either the nurse, family member or ENT consultant, to prevent the patient from exsanguinating from this bleed. If these confederates aid the provider, there must be a detailed discussion, in the debrief, on these missed, critical steps. An additional area of discussion for the debrief includes management of the crash and/or failed airway in one of these scenarios. The complex airway issues of this case may facilitate discussion about key airway teams (ENT, anesthesia and/or surgery) within the training institution and critical junctures when their services should be requested.

During this debriefing we used a 3D anatomic model that included the heart, aortic arch and its branches as well as the trachea and esophagus. This visual aid helped us to review the pathophysiology of a TIAF as well as the mechanism of the Utley maneuver.

**Wrap Up:**

1. **What is the differential diagnosis for bleeding from a tracheostomy site?**^1^,^5^,^9^
  ***-******Immediate Postoperative:*** hemorrhage from direct trauma to site
  ***-******Early Postoperative*** ***(<7 days):*** irritation from suctioning/manipulation, bleeding from surgical site such as tumor, infection/tracheitis, tracheoinnominate artery fistula (TIAF)
  ***-******Late Postoperative*** ***(>7 days):*** granulation tissue, infection at stoma or tracheitis, TIAF
  **-Also consider bleeding from lungs/upper airway and/or gastrointestinal tract**
  **-Consider a bleeding diathesis from coumadin or other blood thinners**
  **-Bleeding from a TIAF usually occurs from 3 days to 6 weeks postoperatively (usually within 7–14 days)**
2. **What are Key Elements of the History for any patient with a Tracheostomy** 9 **?**
  -*When was the tracheostomy performed?*
  *-What was the indication for the tracheostomy (Can the patient be intubated from above)?*
  *-Recent changes/downsizing?*
  *-Recent hospitalization/Infection?*
  *-Anticoagulants?*
3. **General Points Regarding Tracheostomy Tubes** 1,5,7**:**
  -Approximately 110,000 tracheostomies/year are performed in the United States-General emergencies with tracheostomies include obstruction, decannulation and hemorrhage
  -Very few complications with tracheostomy tubes are serious or life-threatening though approximately 1% of tracheostomy complications, such as a TIAF, will be potentially catastrophic and have a >50% mortality.
  -Consider TIAF for ANY tracheostomy-related hemorrhage, especially within 4 weeks of placement
  -If the patient remains stable but has a history of a recent bleed, additional diagnostic options include bronchoscopy, CT Angiography, Traditional Angiography or local exploration in the operating room. All of these modalities should be discussed with the on-call otolaryngologist and/or cardiothoracic surgeon.
  -Tracheostomies are performed more commonly than laryngectomies.
  -A tracheostomy stoma tract is mature after 3–7 days. After this point, the emergency department provider can safely exchange a tube.
  -Tracheostomies which are placed non-emergently are placed inferior to the cricothyroid membrane, between the second and third tracheal rings.
  -Consult with your local otolaryngologist concerning the best tracheostomy tubes to stock in your emergency department
  -It is best to replace a tracheostomy tube with one of similar make and size
  -When replacing a tracheostomy tube, consider exchange over a bougie or other exchange catheter or use an obturator to facilitate placement of the new trach.
4. **What are useful bedside supplies and equipment for care of any tracheostomy patient**^7^**?** Personal protective equipment, pulse oximeter, cardiac monitor, end-tidal waveform capnography (this will not be accurate if using uncuffed tube), multiple sizes of cuffed and uncuffed endotracheal and tracheostomy tubes, bougie or other airway exchange catheter, suction canister with tubing and tracheal suction catheter, bag valve mask (consider peds bag valve mask and laryngeal mask airway in the event that positive pressure ventilation is needed over the stoma), Laryngoscope, Nasopharyngoscope, Suture Removal Kit, Airway Cart, Code Cart
  **What are general indications for tracheostomy tube placement**^5^,^9^,^10^**?**
  -bypass glottic or supraglottic stenosis, airway obstruction (mass), airway collapse secondary to trauma
  -long-term ventilation and assist in removal of respiratory tract secretions (tracheal toilet)
  -facilitate more comfortable weaning from mechanical ventilation
  -protection from aspiration
5. **What are some useful statistics concerning a tracheoinnominate artery fistula** 2 **?**
  -This condition has a 50–70% mortality. Operative repair involves an open, surgical repair or, more recently, endovascular stenting has been introduced as a potentially safer option in a hemodynamically stable patient who is not actively bleeding
  -70% of TIA fistulas will develop in the first 3 weeks but may occur even years later
  -No intervention results in 100% mortality
6. **What are the risk factors for a TIA Fistula**^1^,^2^**?**
  -mispositioned tracheostomy tube (excessive pressure on anterior tracheal wall causes erosion/pressure necrosis)
  -high cuff pressure
  -low-seated tracheostomy (below the 3rd tracheal ring)
  -high-riding innominate artery
  -infection
  -steroids
  -immunosuppression
  -excessive neck movement
7. **Additional points about TIAF** 1 **:**
  -This usually occurs at least 48 hours after tracheostomy procedure.
  -Incidence is 0.7% of all tracheostomy procedures.
  -75% of TIAFs present in the first 3 weeks after surgery.
  -50% of TIAFs present with a sentinel bleed.
8. **Emergent steps for undifferentiated bleeding from tracheostomy site** 5 **?**
9. **Don PPE**
  **Consult ENT/CT Surgery/Respiratory Therapy**
  **Suction the Airway**
  **STAT Reversal agent for any bleeding diathesis. Consider tranexamic acid (TXA)**
  **STOP THE BLEED:**
  -replace uncuffed with cuffed tube and hyperinflate (85% effective)
  -gentle retraction of tube with anterior pressure on trachea
  -Utley Maneuver all the way to the OR

(*some sources state that any bleed >10mL should be assessed by a surgeon, regardless of cause*)

## SIMULATION ASSESSMENT

*Tracheoinnominate Artery Fistula*

Learner: _________________________________________

***Assessment Timeline***

This timeline is to help observers assess their learners. It allows observer to make notes on when learners performed various tasks, which can help guide debriefing discussion.[Table t4-jetem-6-3-s62]

**Critical Actions:**

□ Perform a detailed history and exam of a patient who presents with bleeding from the tracheostomy site. This should include clarification of whether the patient can be intubated from above.
□ Order one or two, large-bore peripheral IVs and bloodwork, including type and screen, complete blood count (CBC), basic metabolic panel (BMP) and coagulation panel.
□ When the rebleed occurs, apply gentle suction and replace the uncuffed tracheostomy tube with a cuffed tracheostomy tube (or cuffed endotracheal tube) of similar size and hyperinflate the cuff.
□ Recognize this is likely a tracheoinnominate artery fistula and emergently consult with otolaryngology, cardiothoracic surgery and the operating room team.
□ If rebleed occurs, gently pull back the tube and exert anterior pressure on the trachea.
□ If rebleed occurs, deflate the cuff and perform the Utley Maneuver. The providers who perform this maneuver must not move their finger until the patient is in the operating room and under the care of the surgical team. If the providers are unable to fit their finger in the stoma, they may reattempt after replacement of the cuffed with an uncuffed tube that is one size smaller.
□ When the patient becomes hemodynamically unstable from recurrent bleeding, the provider must order two units of emergent-release blood and activate a massive transfusion protocol.

**Summative and formative comments:**

**Milestones assessment:** [Table t5-jetem-6-3-s62]
